# Supplementary material for: Structural basis for voltage-sensor trapping of the cardiac sodium channel by a deathstalker scorpion toxin
Source: Nat Commun. 2021 Jan 4;12:128. doi: 10.1038/s41467-020-20078-3 (PMC7782738; doi:10.1038/s41467-020-20078-3)
Supplement: Supplementary file 1 — Supplementary Information [file 41467_2020_20078_MOESM1_ESM.pdf]

SUPPLEMENTARY INFORMATION

Figures and Legends

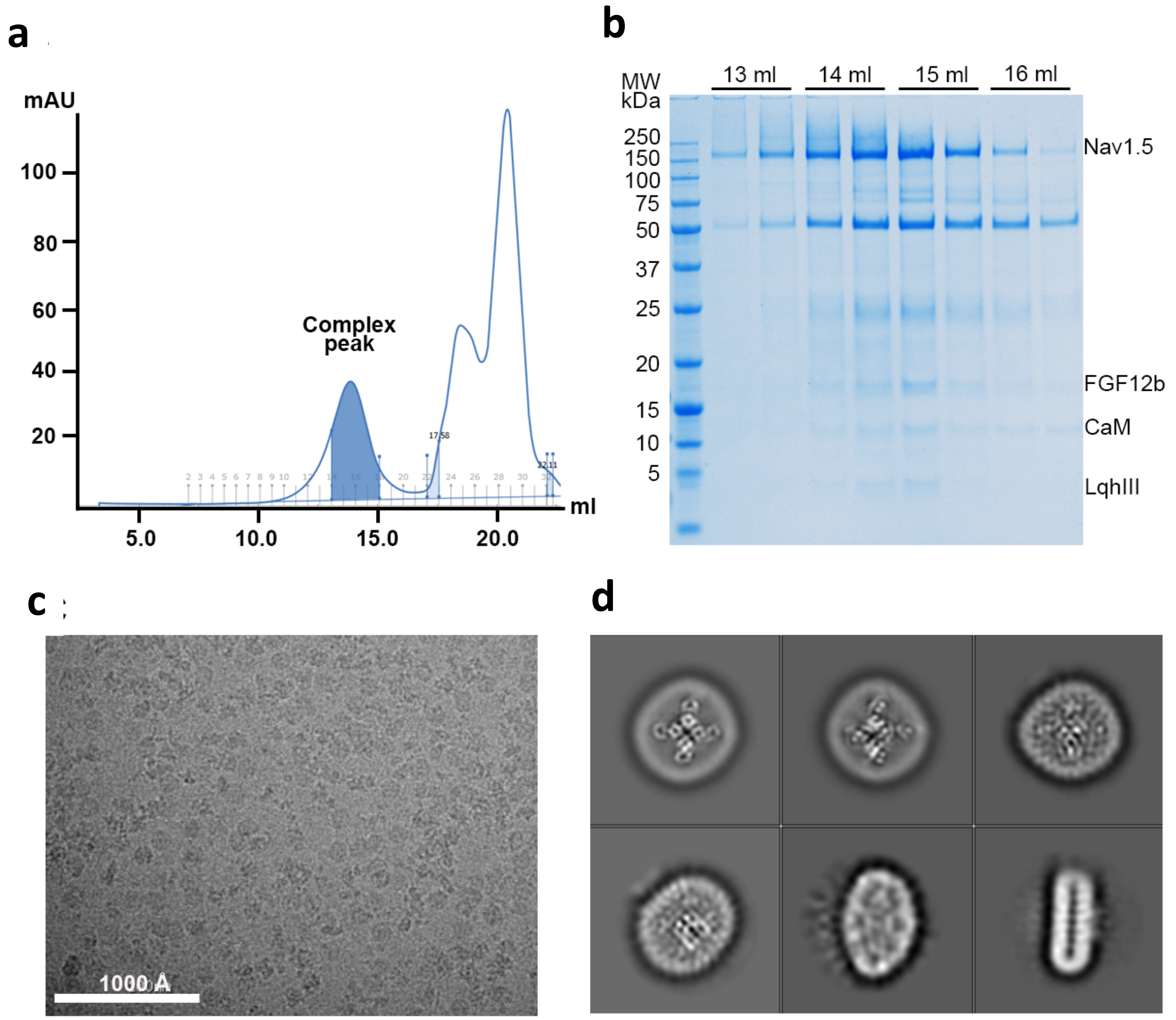

Supplementary Figure 1. Purification and cryo-EM images of rNa$_V$1.5$_C$/LqhIII complex. **a**. Representative size-exclusion chromatography profile of purified rNav1.5$_C$/LqhIII. Peak fractions collected for cryo-EM grid preparation are shown in blue. **b.** SDS-PAGE of the SEC peak fractions stained by Coomassie blue. These results are typical of three independent preparations. **c.** Representative cryo-EM micrograph of rNa$_V$1.5$_C$/LqhIII sample. **d.** Selected reference-free 2D classification averages.

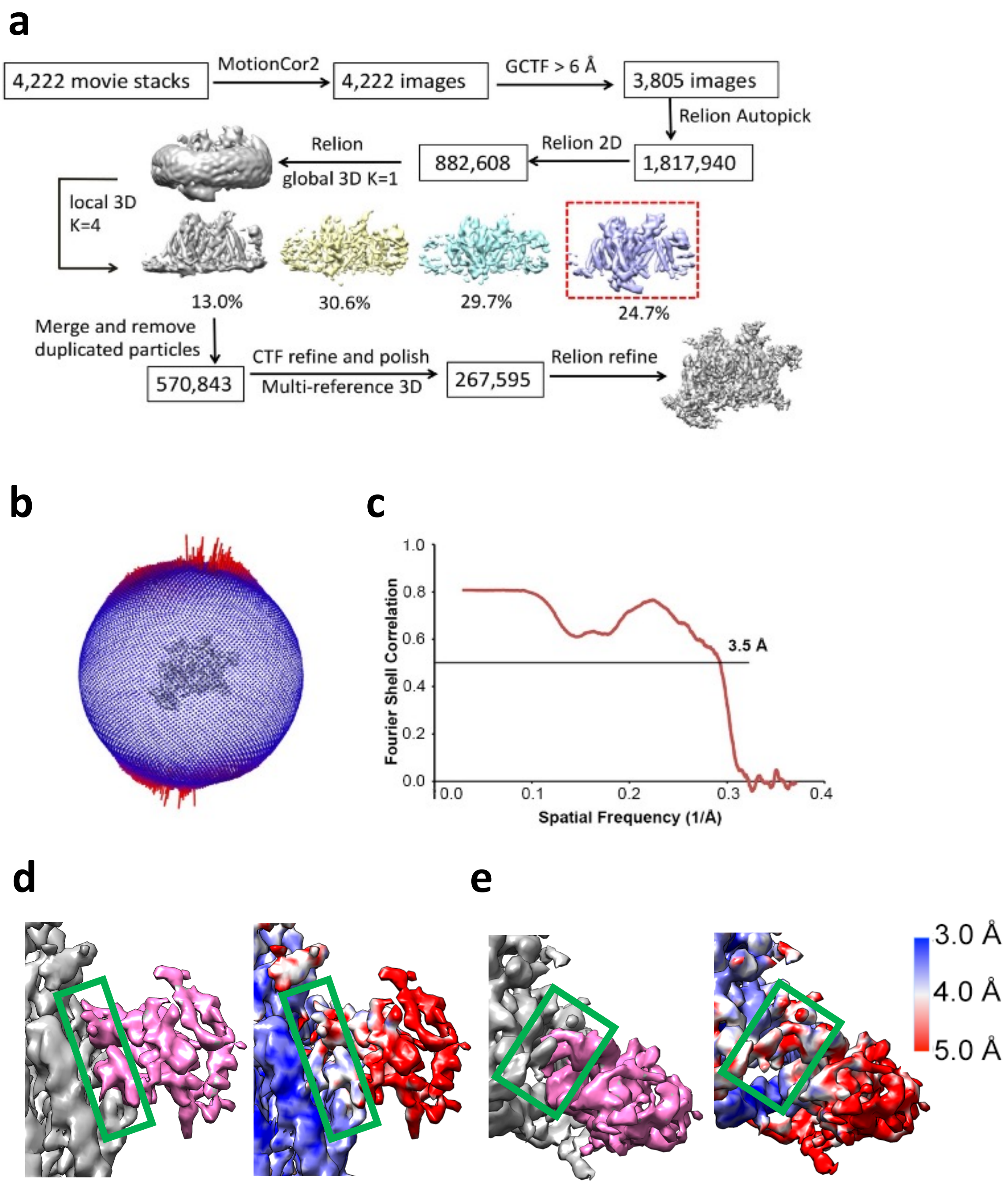

Supplementary Figure 2. Cryo-EM data processing and resolution of the rNa$_V$1.5$_C$/LqhIII complex. **a**. The flowchart of the rNa$_V$1.5$_C$/LqhIII data processing. **b**. Particle angular distribution of the final reconstruction. **c**. The Fourier Shell Correlation curve of the model versus the map used for model refinement. **d**. Expanded side view of the local resolution of the rNa$_V$1.5$_C$/LqhIII interface. **e**. Expanded top view of the local resolution of the rNa$_V$1.5$_C$/LqhIII interface.

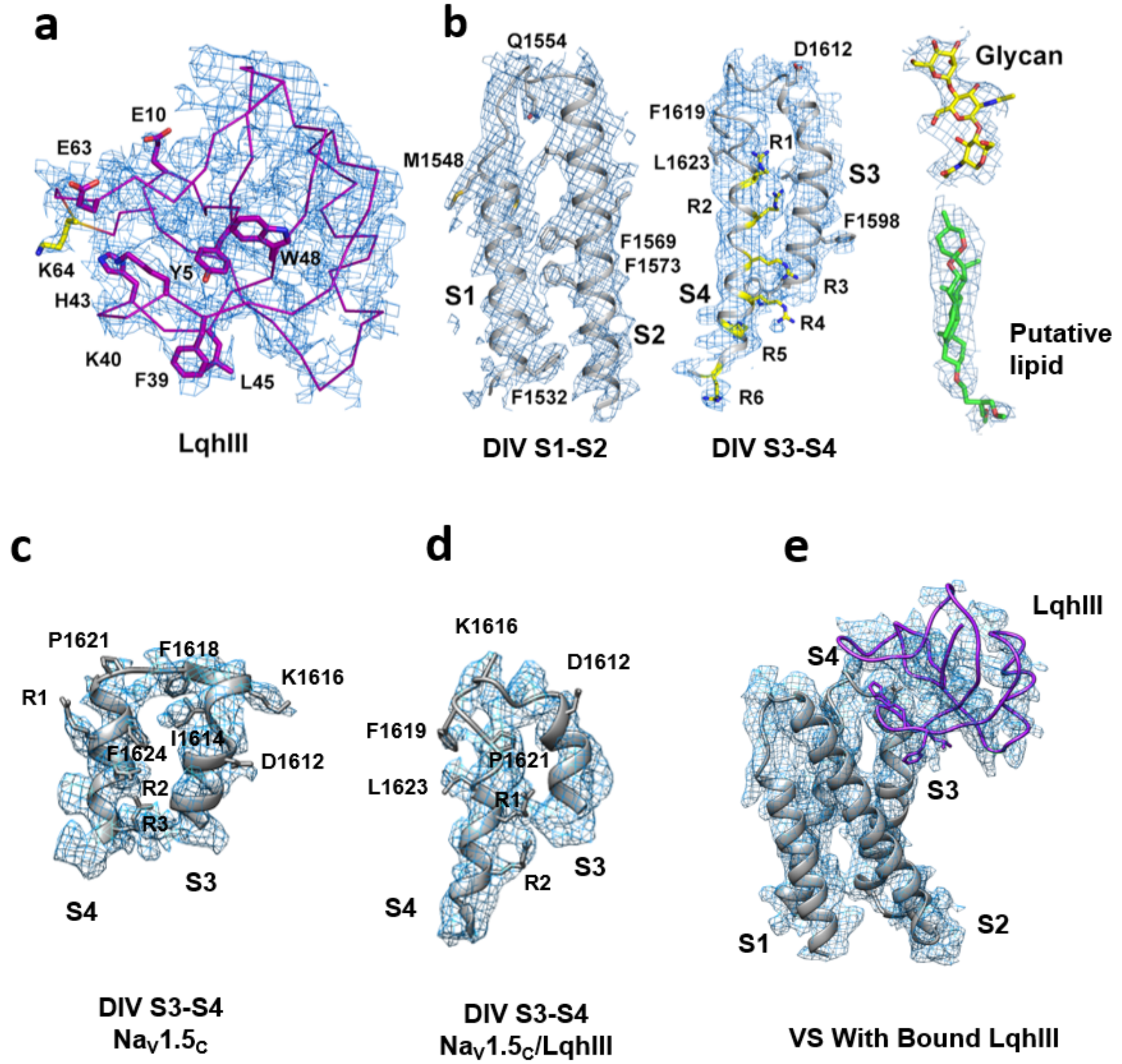

Supplementary Figure 3. Representative EM map for key extracellular components of the rNa$_V$1.5$_C$/LqhIII complex. **a**. The EM density for LqhIII. **b**. The EM density for *D*IV S1-S2, *D*IV S3-S4, N329 linked glycan, and a putative lipid (modeled as the detergent GDN in green sticks) inside the activation gate, respectively. Key residues are shown in sticks. **c**. EM density for the S3-S4 linker in rNa$_V$1.5$_C$. **d**. EM density for the S3-S4 linker in Na$_V$1.5c/LqhIII. **e**. EM density for the complex of Na$_V$1.5$_C$/S3-S4 and LqhIII.

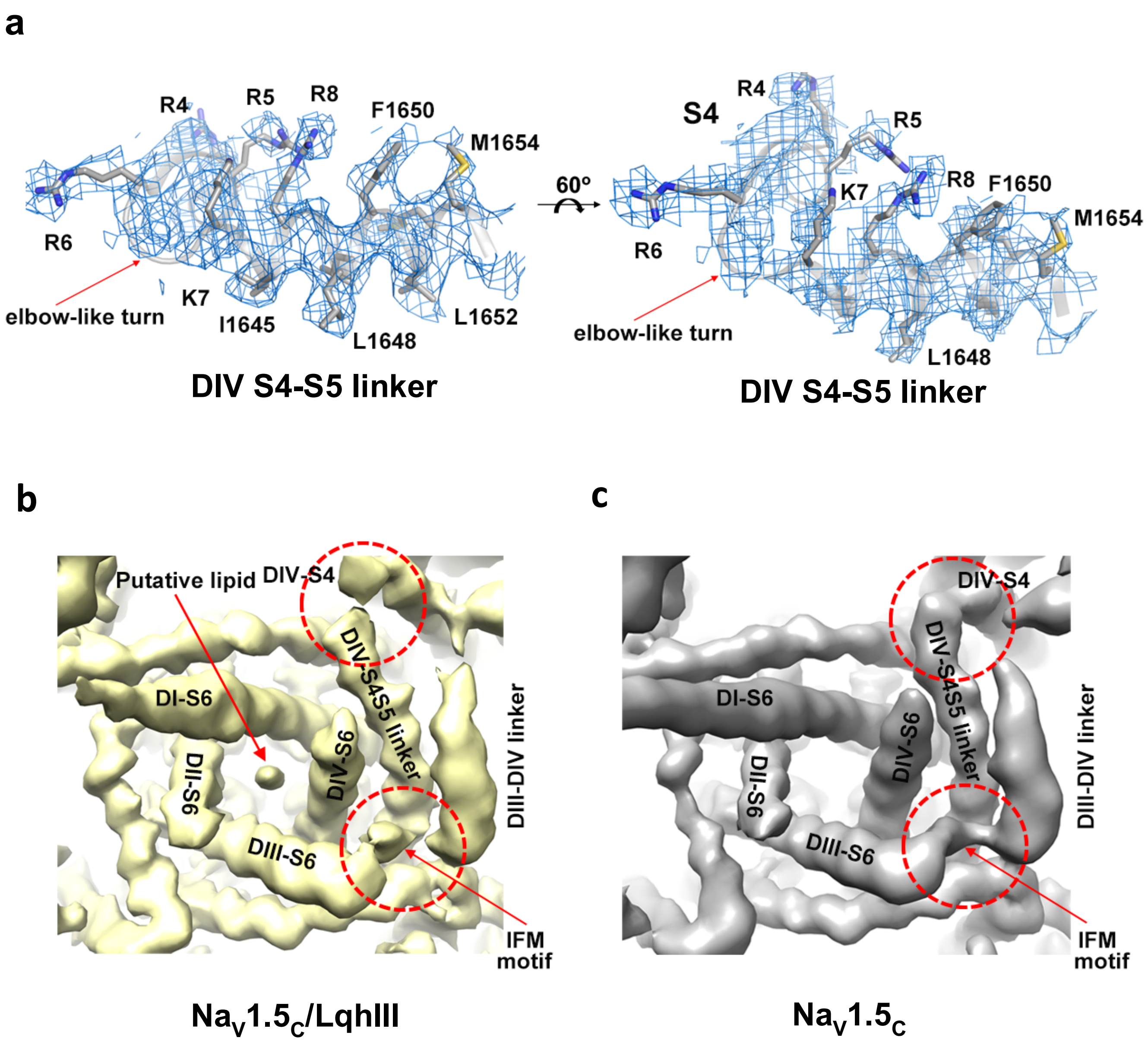

Supplementary Figure 4. Representative EM map for key intracellular components of the rNa$_V$1.5$_C$/LqhIII complex. **a**. EM density for the rNa$_V$1.5$_C$/LqhIII *D*IV S4-S5 linker. (**b-c**) The unsharpened EM density comparison between rNa$_V$1.5$_C$/LqhIII (yellow) and rNa$_V$1.5$_C$ without toxin (grey) in a bottom (intracellular) view of the intracellular activation gate and inactivation gate. Red dash circles indicated the areas of conformational change in the two maps.

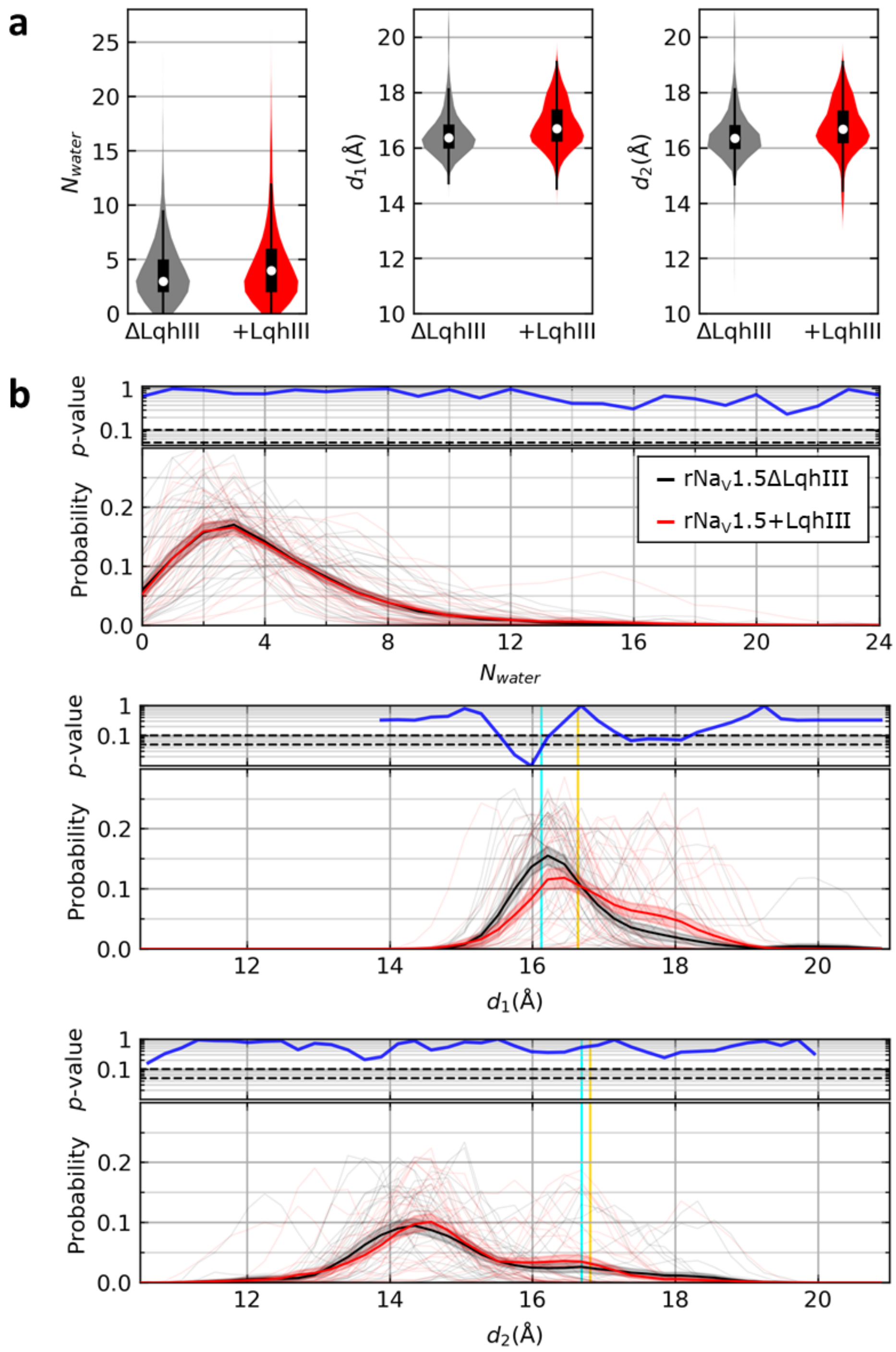

a
b
N_water
ΔLqhIII
+LqhIII
d1(Å)
d2(Å)
p-value
Probability
rNaV1.5ΔLqhIII
rNaV1.5+LqhIII

Supplementary Figure 5. Significance of changes in size and hydration of the intracellular activation gate. a. Differences in mean values of hydration and diagonal distances of the intracellular activation gate due to toxin binding. Violin plots of means and s.e.m. Left, the number of water molecules in the intracellular gate (N_{water}). Middle, d_1 (*D*I-*D*III). Right, d_2 (*D*II-*D*IV) for simulations of rNa$_V$1.5$_C$ with (red) or without (grey) LqhIII. The median (white dot), quartiles (thick black line), and interquartile ranges (thin black line) are shown. *p*-values were calculated from a two-sided Welch's t-test which indicated significant differences in the means of the distributions between simulations: Left: N_{water} = 3.958 ± 0.017 for Na$_V$1.5$_C$; N_{water} = 4.307 ± 0.019 for rNa$_V$1.5$_C$/LqhIII, p=2x10^{-42}. Middle: d_1 = 16.520 ± 0.005 for rNa$_V$1.5$_C$; d_1 = 16.810 ± 0.001 for rNa$_V$1.5$_C$/LqhIII; p=0.001. Right: d_2 = 16.465 ± 0.005 for rNa$_V$1.5$_C$; d_2 = 16.744 ± 0.005 for rNa$_V$1.5$_C$/LqhIII; p=7x10^{-300}. Mean and s.e.m. Time frames spread across 30 independent simulations were used for significance testing (n=29,026 frames for rNa$_V$1.5$_C$ and n=29,899 frames for rNa$_V$1.5$_C$/LqhIII). **b.** Changes in hydration and diagonal distances of the intracellular activation gate due to LqhIII binding. Top. *p*-values calculated from a two-sided Welch's t-test for the probability of N_{water} in n=30 independent MD simulations of the cryo-EM rNa$_V$1.5$_C$ structure with or without LqhIII. Toxin-binding insignificantly changes the hydration of the gate. Significance levels of 0.1 and 0.05 are indicated as black and dashed lines. Bottom. The thick line with shading is the average probability distribution of N_{water} and the thin and transparent lines are distributions from 30 independent replicas for simulations with (red) or without (black) LqhIII. Shading represents s.e.m. In the presence of the toxin, d_1 is significantly larger, as indicated by both a significant decrease in the probability of d_1 = 15.8 Å to 16.2 Å ([d_1 (Å), *p*] = {(15.8, 2×10^{-2}), (16.0, 1×10^{-2}), (16.2, 9×10^{-2})}) and increase in the probability of d_1 = 17.4 Å to 18.0 Å ([d_1 (Å), *p*] = {(17.4, 7×10^{-2}), (17.6, 8×10^{-2}), (17.9, 8×10^{-2}), (18.0, 8×10^{-2})}). The *p*-values and average probability distribution of diagonal distance, d_2, between S6 helix tails of *D*II and *D*IV. No significant change is observed. d_1 and d_2 of the reference cryo-EM structures of rNa$_V$1.5$_C$ (PDB ID: 6UZ3; cyan) and rNa$_V$1.5$_C$/LqhIII (yellow) are indicated by colored vertical lines.

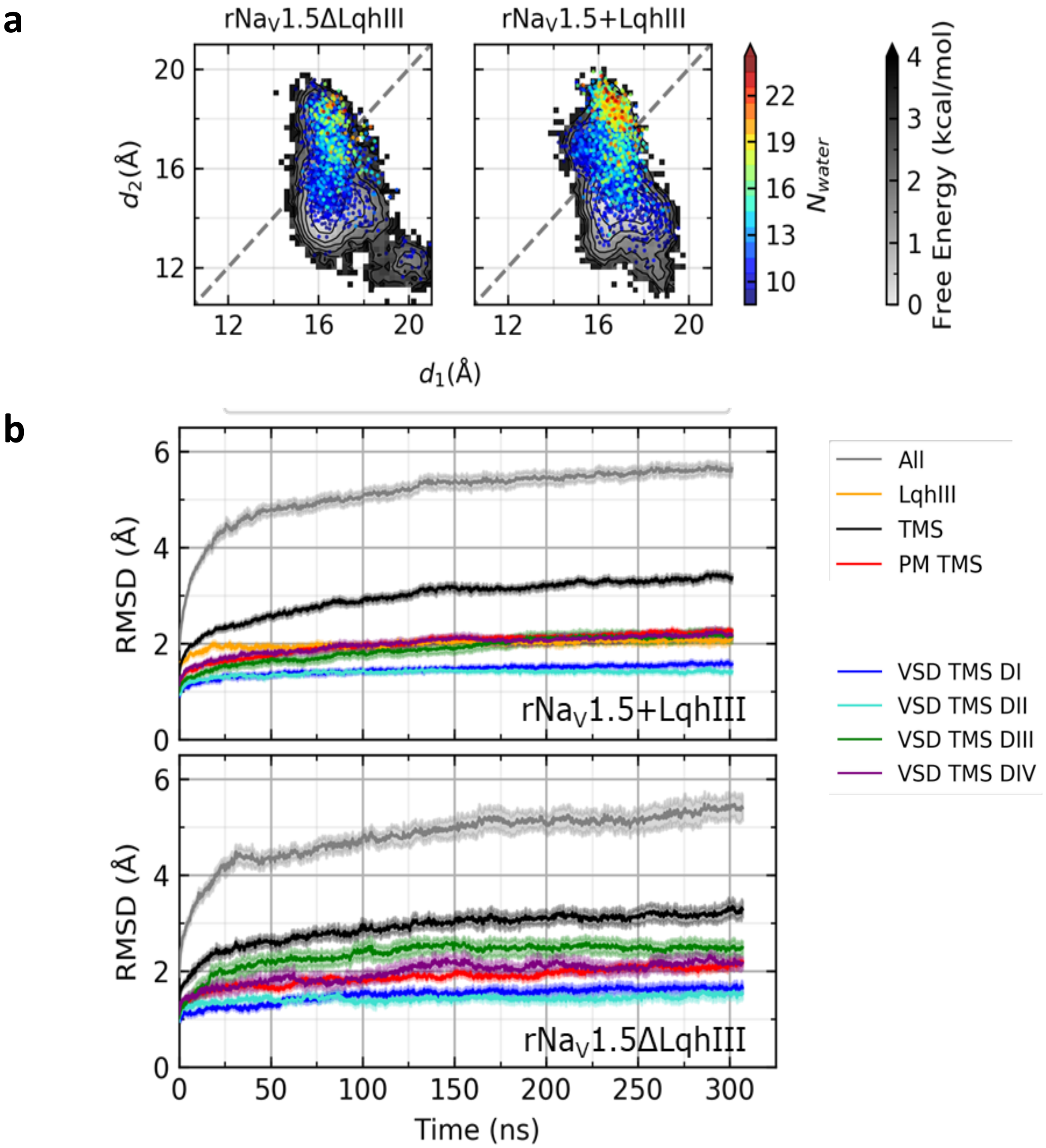

Supplementary Figure 6. Hydration and time evolution of diameter in the intracellular activation gate. a. Hydration of the intracellular activation gate at dilated conformations. Free energy distribution of diagonal distances (grey-scale) between S6 helix tails (d_1: DI-DIII, d_2: DII-DIV); same as Figure 6. d_1 and d_2 conformations corresponding to $N_{water} \geq 9$ are shown as circles colored according to N_{water}. Conformations with $N_{water} \geq 15$ are most often found when $d_2 \geq d_1 > 16$ Å. **b.** Time evolution of the protein root-mean-square deviation (RMSD). The average RMSD was calculated over time relative to the initial cryo-EM $rNa_V1.5_C$/LqhIII structure for simulations with (top) or without (bottom) the toxin. The RMSD of backbone Cα atoms for all residues (grey), LqhIII only (orange), transmembrane segments (TMS) of $rNa_V1.5_C$ (black), as well as TM segments from the pore module (PM; red) and voltage sensing domains (VS) from *D*I (blue), *D*II (cyan), *D*III (green), and *D*IV (purple) are shown. Based on PM, TMS, and LqhIII RMSD values, simulations equilibrate at approximately 100 ns. Time frames spread across 30 independent simulations of $rNa_V1.5_C$/LqhIII with or without the toxin were used for analyses (n=29,026 frames for $rNa_V1.5_C$ and n=29,899 frames for $rNa_V1.5_C$/LqhIII).

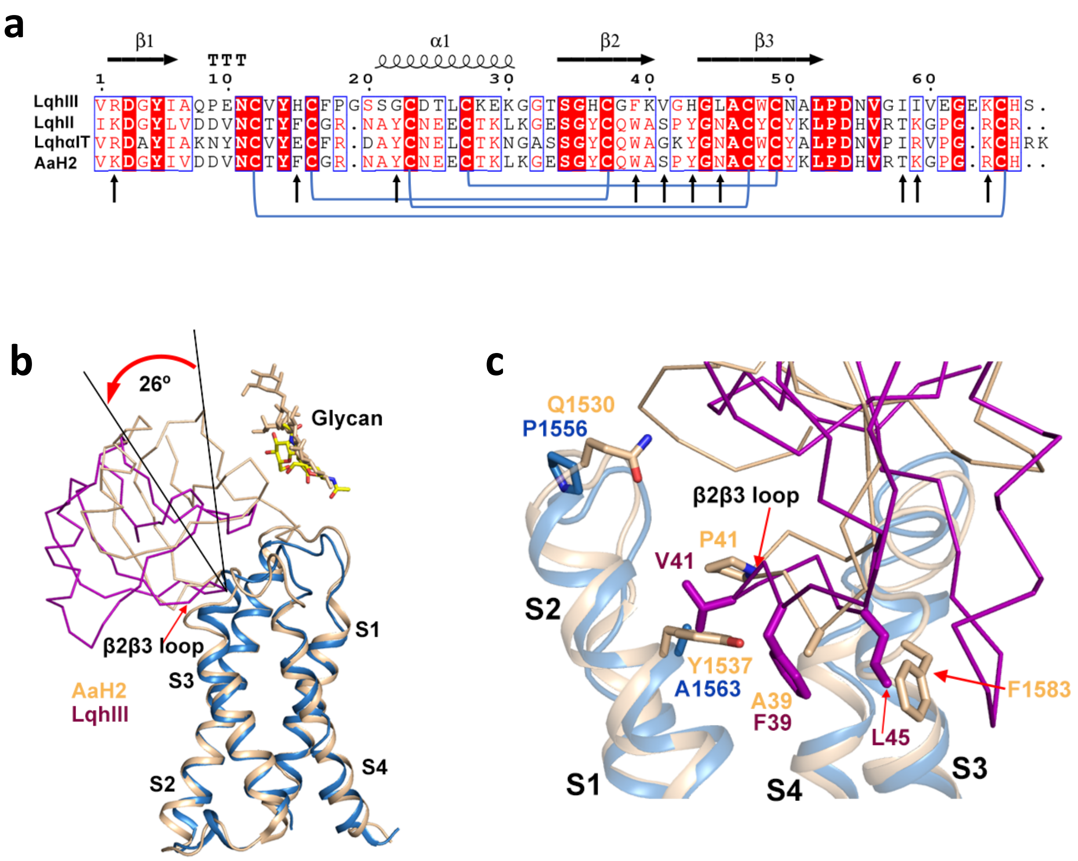

Supplementary Figure 7. Comparison of the structure of the Na$_V$1.7/Na$_V$Pas/AaHII complex to the structure of the rNa$_V$1.5$_C$/LqhIII complex. **a**. Sequence alignment among α-scorpion toxins. Disulfide-bond forming Cys were connected by blue lines. Key residues from site-directed mutagenesis studies were indicated with black arrows. **b**. Superposition of rNa$_V$1.5$_C$ *D*IV-VS/LqhIII and Na$_V$1.7 *D*IV-VS/AaHII. Na$_V$1.7 *D*IV-VS/AaHII was colored in wheat. **c**. Comparison of the binding positions in the rNa$_V$1.5$_C$/LqhIII complex (purple) and the Na$_V$Pas-Na$_V$1.7-*D*IV/AaHII complex (wheat). Key residues shown in sticks.

Table S1. Cryo-EM data collection, refinement and validation statistics

	$rNa_v1.5_C$/LqhIII (EMDB code: EMD-22621) (PDB code: 7K18)
**Data collection and processing**	
Magnification	130,000
Voltage (kV)	300
Electron exposure (e-/Å^2)	60
Defocus range (μm)	1.2 – 2.8
Pixel size (Å)	1.056
Symmetry imposed	C1
Initial particle images (no.)	1,817,940
Final particle images (no.)	267,595
Map resolution (Å)	3.34
FSC threshold	0.143
Map resolution range (Å)	3.0 – 5.0
**Refinement**	
Initial model used (EMDB code)	
Model resolution (Å)	3.5
FSC threshold	0.5
Model resolution range (Å)	3.0 – 5.0
Map sharpening *B* factor (Å^2)	-86
Model composition	
Non-hydrogen atoms	9973
Protein residues	1195
Ligand	25
B factors (Å^2)	
Protein	59.0
Ligand	53.1
R.m.s. deviations	
Bonds (Å)	0.004
Angels (°)	0.758
Validations	
MolProbity score	2.75
Poor rotamers (%)	0.8
Ramachandran plot	
Favored (%)	93.99
Allowed (%)	5.76
Disallowed (%)	0.25

Table S2. Molecular Dynamics Simulation Parameters

MD parameters used for each pre-equilibration and production step. The ensemble (ENS), time integration step, and total time/replica are indicated. Berendsen (B) (1) or Nosé-Hoover (NH) (2, 3) thermostats and B or Parrinello-Rahman (PR) (4, 5) barostats with their respective coupling times constants (CTC) are shown. Protein backbone and sidechain position restraints as well as lipid position and dihedral restraints are also specified.

				Temperature (T)			Pressure (P)			Restraints ($kJ/mol/nm^2$)			
Step	ENS	Time Step (fs)	Total Time (ns)	T (K)	Thermo-stat	T CTC	P (bar)	Baro-stat	P CTC	Protein Backbone	Protein Sidechain	Lipid Position	Lipid Dihedral
1	NVT	1	0.025	300	B	1 ps	---	---	---	4000	2000	1000	1000
2	NVT	1	0.05	300	B	1 ps	---	---	---	2000	1000	1000	400
3	NPT	2	10	300	B	1 ps	1	B	5 ps	1000	500	400	200
4	NPT	2	0.1	300	B	1 ps	1	B	5 ps	500	200	200	200
5	NPT	2	0.1	300	B	1 ps	1	B	5 ps	200	50	40	100
6	NPT	2	0.1	300	B	1 ps	1	B	5 ps	50	0	0	0
7	NPT	2	300-400	300	NH	1 ps	1	PR	5 ps	0	0	0	0

References

1. Berendsen, H. J. C., Postma, J. P. M., Gunsteren, W. F. van, DiNola, A., and Haak, J. R. (1984) Molecular dynamics with coupling to an external bath. *J Chem Phys*. **81**, 3684–3690

2. Hoover, W. G. (1985) Canonical dynamics: Equilibrium phase-space distributions. *Phys Rev A*. **31**, 1695–1697

3. Nosé, S. (2006) A molecular dynamics method for simulations in the canonical ensemble. *Mol Phys*. **52**, 255–268

4. Parrinello, M., and Rahman, A. (1980) Crystal Structure and Pair Potentials: A Molecular-Dynamics Study. *Phys Rev Lett*. **45**, 1196–1199

5. Nosé, S., and Klein, M. L. (2006) Constant pressure molecular dynamics for molecular systems. *Mol Phys*. **50**, 1055–1076
